# Chrono-Nutrition: Circadian Rhythm and Personalized Nutrition

**DOI:** 10.3390/ijms24032571

**Published:** 2023-01-29

**Authors:** Marica Franzago, Elisa Alessandrelli, Stefania Notarangelo, Liborio Stuppia, Ester Vitacolonna

**Affiliations:** 1 Department of Medicine and Aging, School of Medicine and Health Sciences, G. d’Annunzio University, 66100 Chieti, Italy; 2 Center for Advanced Studies and Technology, G. d’Annunzio University, 66100 Chieti, Italy; 3 Department of Psychological Health and Territorial Sciences, School of Medicine and Health Sciences, G. d’Annunzio University, 66100 Chieti, Italy

**Keywords:** gut microbiome, clock genes, epigenetics, gene–diet interaction, nutrigenetics, personalized nutrition, chronodisruption

## Abstract

The human circadian system has a period of approximately 24 h and studies on the consequences of “chornodisruption” have greatly expanded. Lifestyle and environmental factors of modern societies (i.e., artificial lighting, jetlag, shift work, and around-the-clock access to energy-dense food) can induce disruptions of the circadian system and thereby adversely affect individual health. Growing evidence demonstrates a complex reciprocal relationship between metabolism and the circadian system, in which perturbations in one system affect the other one. From a nutritional genomics perspective, genetic variants in clock genes can both influence metabolic health and modify the individual response to diet. Moreover, an interplay between the circadian rhythm, gut microbiome, and epigenome has been demonstrated, with the diet in turn able to modulate this complex link suggesting a remarkable plasticity of the underlying mechanisms. In this view, the study of the impact of the timing of eating by matching elements from nutritional research with chrono-biology, that is, chrono-nutrition, could have significant implications for personalized nutrition in terms of reducing the prevalence and burden of chronic diseases. This review provides an overview of the current evidence on the interactions between the circadian system and nutrition, highlighting how this link could in turn influence the epigenome and microbiome. In addition, possible nutritional strategies to manage circadian-aligned feeding are suggested.

## 1. Introduction

The circadian rhythms (circa = around and dies = one day), occurring at central and local levels by the involvement of clocks within several peripheral tissues [1,2], regulate many behavioral and biochemical processes through the day/night cycle [2]. In addition, the core circadian clock machinery can be modulated by energy/nutrient input, thus pointing to the important role of energy metabolism [3,4]. In this context, a reciprocal and complex interconnectivity between the circadian clock system and metabolism has been identified; this relationship makes it likely that perturbations in one system affect the other. Circadian desynchrony typical of modern societies and triggered by several chrono disruptors (such as shift work, stress, jetlag, sleep disruption) can impair human health, leading to an increased risk of metabolic diseases. Diet is one of the synchronizers of our clock mechanisms, thus abnormal feeding times can lead to the separation of environmental oscillators from the central pacemaker inducing unhealthy consequences.

From a nutrigenetics point of view, several genetic variants in circadian-related genes, interacting with dietary intakes and obesogenic behaviors, can influence the individual response to diet, suggesting that chronobiology should be taken into account in nutritional practice [5,6,7,8]. On the other hand, the circadian rhythm is optimized for feeding in the light phase [9], thus nutritional input improves circadian function by coordinating the peripheral and central clocks.

Additionally, epigenomes and gut microbiomes show diurnal rhythms. The epigenetic mechanisms play an important role in the regulation of the molecular clock machinery transcription, and clock-controlled genes, gut microbiota (GM), and microbial metabolites are known to mediate the effects of disruptions of circadian rhythms on human health. Therefore, new opportunities have risen from recent findings on a dynamic crosstalk among diet–biological rhythm-omics [10,11,12]. The effects of dietary components on health outcomes are widely explored, nevertheless the complex relationship between meal timing and the circadian machinery is still under investigation. Recently, chrono-nutrition has emerged as a new area of research studying the impact of the timing of eating on the well-being of an organism. In particular, the modification of the cycle between periods of eating and fasting has been associated with a predisposition to nutrition-related diseases including obesity, type 2 diabetes (T2DM), and cardiovascular disease (CVD). This review provides an overview of the current evidence on the genetic and environmental factors inducing molecular clock disruption with relevance to the onset of non-communicable diseases (NCDs). It also explores the interactions between the circadian system and diet, highlighting how this link in turn influences the epigenome and microbiome. Finally, it suggests possible nutritional strategies to manage circadian-aligned feeding.

## 2. Circadian Rhythms

The circadian system, composed of a set of interconnected clock oscillators located in the suprachiasmatic nuclei (SCN) of the hypothalamus and in some metabolically active peripheral organs, regulates the behavioral and physiological daily rhythms of sleep/wake, fasting/feeding and catabolic/anabolic cycles, body temperature, and endocrine functions [13].

Although the hypothalamic SCN is considered as the dominant circadian pacemaker, most peripheral organs and tissues can express circadian oscillations in isolation even if they interact with each other and with the system as a whole. The synchronization of peripheral clocks plays an important role in ensuring the temporally coordinated physiology [14]. Several factors are expressed and secreted following circadian stimuli, inducing functional modifications including the following: (i) glucose tolerance peaks during daylight and is lower during the night/dark cycle, (ii) melatonin drops at 7:00 and rises at 20:00, (iii) cortisol rises at 8:00, (iv) sleep deepens at 1:00, (v) body temperature rises at 3:00 [15].

At the molecular level, circadian oscillations are generated by a complex array of genes known as “clock genes”, including the clock circadian regulator (*CLOCK*), aryl hydrocarbon receptor nuclear translocator-like (*BMAL1*, also known as *ARNTL*), period circadian regulators (*PER1* and *PER2*), and cryptochrome circadian regulators (*CRY1* and *CRY2*). Proteins encoded by these genes play a crucial role in the regulation of the circadian rhythmicity.

**Figure 1:**
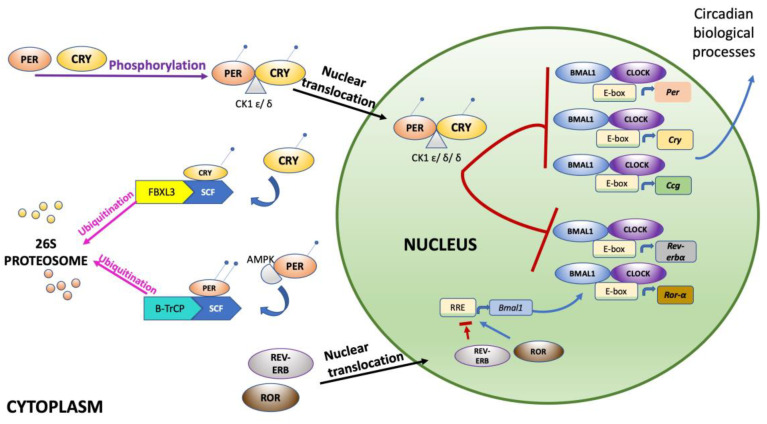
Molecular mechanisms controlling the circadian rhythm. CLOCK and BMAL1 regulate the expression of numerous genes including Per family (*Per1-3*), Cry family (*Cry1-2*), nuclear receptor family (*Rev-erbα* and *Rorα*), and many downstream target genes known as clock-controlled genes (*Ccg*). CRY and PER proteins translocate to the nucleus to form a negative-feedback repressor complex of CLOCK/BMAL1 transcriptional activity. Another feedback loop, driven by CLOCK:BMAL1, involves Rev-erbα and Rorα to regulate Bmal1 transcription. At a post-transcriptional level, SCF (Skp1-Cullin-F-box protein) E3 ubiquitin ligase complexes regulate PER and CRY proteins’ stability by recognizing specific targets and directing their polyubiquitination. Finally, their degradation is regulated by the 26S proteasome complex.

The molecular clock is based on a transcriptional autoregulatory feedback loop characterized by the activation of BMAL1 and CLOCK, which positively regulate the expression of their target PER and CRY at the beginning of the cycle (Figure 1). The negative-feedback repressor complex PER/CRY translocates into the nucleus to suppress activity of BMAL1/CLOCK [13,16]. This feedback cycle has a genetically determined period length of approximately 24 h and is synchronized to the environment mostly via light. Furthermore, CLOCK-BMAL1 also drives another dozen downstream target genes known as clock-controlled genes. The circadian network is a complex finely regulated system: the stability of the PER and CRY proteins is controlled by SCF (Skp1-Cullin-F-box protein) E3 ubiquitin ligase complexes. In addition, the phosphorylation of the PER and CRY proteins are triggered by the kinases casein kinase 1ε/δ (CK1ε/δ) and AMP kinase (AMPK), respectively, inducing the polyubiquitination by their respective E3 ubiquitin ligase complexes, which in turn activate the 26S proteasome complex to degrade the PER and CRY proteins [17].

The auxiliary feedback loop involving *Rev-erbα* represses the transcription of *Bmal1*, triggering an antiphase oscillation in *Bmal1* gene expression [18]. As described in the paragraphs below, under circadian misalignment, the central and peripheral signals conflict due to the misalignment of light/dark and feeding/fasting cycles, promoting risk-associated metabolic patterns and chronic diseases such as obesity [19].

## 3. Chronotype

Several studies in chronobiology supported the importance of circadian rhythms in metabolic regulation [15]. Although the circadian system is entrained to an external light–dark cycle with a period of approximately 24 h, there are interindividual circadian preferences influencing behavioral patterns, defined as chronotypes. Chronotype is a biological characteristic leading interindividual differences in the circadian phase relative to the light–dark cycle. There are three general categories of chronotypes usually divided by the terms: (i) “morning”, (ii) “evening”, and (iii) “intermediate” types [20]. Morning types prefer activities at the beginning of the day, and evening types prefer main activities in the late afternoon or evening, and at their extreme this may be shifted by about 2–3 h in circadian oscillations [21]. The intermediate chronotype occupies an intermediate position between the morning and evening types. Several studies described the different features between extreme groups in circadian rhythmicity [22,23,24]. Regarding the extreme chronotypes, morning types are characterized by a phase advance in the peak of body temperature and alertness, in the sleep–wake cycle, and in performance compared with evening types [21,25]. Some studies demonstrated that the evening chronotype is associated with irregular eating and meal skipping, particularly breakfast skipping as well as being related to a lower intake of fruits and vegetables and a higher intake of energy drinks and fat, suggesting long-term consequences on cardiometabolic health [26]. In this view, the evening chronotype has been also correlated with the risk of a variety of conditions, including metabolic dysfunction, diabetes, gastrointestinal/abdominal disorders, psychiatric symptoms, as well as with some cardiovascular risk factors (i.e., higher rates of smoking and overweight/obesity) when compared with the morning chronotype [22,27,28]. 

The later chronotype has been associated with poorer glycemic control in T2DM patients [29] as well as the increased eveningness being related to an increased risk of all-cause mortality over 6.5 years [28]. Moreover, an association between eveningness preference and eating disorders (EDs) has been also suggested [30,31] since it has been shown that the percentage of evening types in the ED group was twice that of the controls [25]. 

The above-described data suggest that chronotype may be predictive of disease outcomes, highlighting a possible relevant role of the circadian system in metabolic regulation. In this view, the relationships between circadian misalignment and metabolic diseases in adolescence, which is considered a vulnerable period for obesity, have also been investigated. Weiss and colleagues [32] reported that adolescents sleeping less than eight hours consumed a higher proportion of calories from fats compared to those with a nocturnal sleep of more than eight hours. Later-chronotype young adolescents are at risk of increased BMI and poorer dietary behaviors with a higher frequency of consuming unhealthy snacks, night-time caffeine consumption, and inadequate daily intake of fruit and vegetables [33]. In this view, future studies are needed to explore the role of the circadian system in the regulation of body weight and metabolism in younger populations.

## 4. Chronodisruptions

The role of circadian disruption in the susceptibility to NCD has received an increasing amount of attention. Circadian clock mutant mice regarding *Bmal1*, *Clock*, or *Rev-erb* genes showed reduced insulin secretion, impaired glucose tolerance, defects in the proliferation and size of pancreatic islets, abnormal lipid profiles, fatty liver, and hyperglycaemia, demonstrating a potential link between clock gene dysregulation and obesity, diabetes, and metabolic syndrome (MetS) [2,34,35,36,37]. Apart from abnormalities in the molecular circadian clock observed in mice, environmental and lifestyle factors which are considered “exposures” or “effectors” (chrono disruptors), can predispose to individual circadian disruption centrally or peripherally, thereby paving the way for chronic disorders. Thus, chronodisruption is defined by Erren et al. as a “disturbance of the circadian organization of physiology, endocrinology, metabolism, and behaviour” [38]. Chronodisruption is common and rising worldwide resulting from our personal modern lifestyles, including excessive energy consumption, irregular times of food consumption, sleep disturbances, and shift work. 

It was also reported that prolonged short sleep durations and/or poor sleep quality with circadian misalignment are correlated with metabolic dysfunctions, including obesity, T2DM, and hypertension [39,40,41,42,43], as well as with decreased leptin, increased appetite, and insulin resistance [42,44]. The night shift work is considered one of the negative components strongly correlated with circadian disruption that induces adverse health effects such as metabolism abnormalities [45]. A meta-analysis of 28 studies demonstrated that shift work had a negative impact on the development of overweight and obesity (Odd Ratio OR = 1.23 (95% confidence interval = 1.17–1.29)) [46]. Scheer et al. [47] examined the deleterious effects of jetlag or shift work, exposing some subjects to a light–dark cycle lengthened to 28 h, out of synchrony with the endogenous clock in which the melatonin and body temperature rhythm free runs with a ~24-h period. The authors showed that this experimental short-term circadian misalignment following a 10-day laboratory protocol increased postprandial glucose, insulin, and mean arterial pressure, as well as induced the decreases in leptin and sleep efficiency, and the complete inversion of the cortisol profile across the behavioral cycle.

Regarding the young population, the effects of sleep duration and social jetlag have been investigated in both adolescents and adults showing that social jetlag was a better predictor of overweight/obesity [48]. Similarly, Chaput and Tremblay [39] also showed that short sleep duration was significantly associated with increased central adiposity in children. Beebe et al. [49] reported that chronic sleep restriction in adolescents (age 14–16 years) caused an increased consumption of foods with high glycemic index (particularly desserts and sweets) and a trend toward more calories and carbohydrates. A randomized crossover design with two experimental conditions based on three consecutive nights of short sleep (4 h/night) or long sleep (9 h/night) duration investigated the effects of sleep deprivation on markers of glucose metabolism in normal-weight adolescents (mean age 16.8 years) [50]. This study showed increased levels of fasting insulin and insulin resistance and 24% reduced epinephrine following sleep restriction compared to adequate sleep duration opportunity [50]. All these data confirmed that the chrono disruptors contribute to various obesity markers.

## 5. Chrono-Nutrition 

Modern lifestyle habits are characterized by being more often in a postprandial state, exposure to unhealthy diets, being sedentary with prolonged sitting times, irregular times of eating, skipping meals, chronic psychological stress, emotional eating, and food consumption late at night [51,52]. 

Therefore, modern lifestyle habits trigger a vicious cycle, in which an obesity-causing unhealthy lifestyle results in disrupted circadian rhythms, which in turn leads to obesity. Several studies showed a beneficial effect of dietary regimens which are based on an availability of food only at discrete windows of time within the daily cycle [15]. A growing body of evidence suggests that these strategies can delay and often reverse the symptoms associated with metabolic disorders, reducing insulin resistance and increasing glucose tolerance [53,54,55,56].

These dietary approaches, through the manipulation of the feeding–fasting cycle, can encompass (i) sustained periods of chronic energy restriction, characterized by a reduction by up to 40% in daily energy intake, but meal frequency and timing remain unchanged; (ii) intermittent fasting, in which one day or more days of fasting are interspersed with normal ad libitum eating patterns; and (iii) chrono-nutrition in which food consumption is restricted to specific times of day. In this context, feeding time restricted (FTR), requiring a consistent daily eating window, is a form of chrono-nutrition. In FTR, the daily eating duration, that is, the time between the first and last energy intake, is typically reduced from a 12–14 h day to <10 h day [8,55,57,58].

The concept of chrono-nutrition was developed in 1986 by Dr. Alain Delabos [59]. It is a nutritional regimen that follows our biological clock, which in turn is marked by changes in metabolism that occur throughout the day. Since later meal timings and irregular eating, which are not in line with the biological clock, are associated with increased adiposity, T2DM, and cardiometabolic risk factors [60,61,62], chrono-nutrition is based on three different dimensions of eating behavior, including timing, frequency, and regularity [26,63,64,65].

Among several dietary strategies available, the chrono-nutritive therapy based on chronobiology is characterized by eating most calories and carbohydrates at lunch time and in the early afternoon, avoiding late evening dinner. In this view, in addition to the amount and content of macronutrients and micronutrients, the timing of food intake during light time vs. evening vs. night is critical for the well-being of an organism and could represent an interesting strategy to maintain metabolic health or to promote weight loss [66].

## 6. Clock Genes Variants

The effect of chronotype combined with the genotypes of several clock genes through eating time has been also investigated. Several single nucleotide polymorphisms (SNPs) in circadian-related genes have been associated with the susceptibility to obesity, CVD, and MetS, as well as gene-diet interactions being described for some of these genetic variants [67,68,69,70]. 

In this view, the *CLOCK* rs4580704 *C* > *G* is one of the most relevant SNPs. The carriers of the minor allele (*G*) in this *CLOCK* variant showed a lower weight, 31% decreased diabetes risk, and 46% lower risk of hypertension than non-carriers [71]. The SNP entitled 3111 *T*/*C* (rs1801260), a substitution of a thymine to cytosine in the 3′-flanking region in the *CLOCK* gene, was associated with eating behaviors related to late eating and evening types, higher BMI, higher ghrelin, and lower GLP-1 levels, thus influencing the susceptibility to obesity and related diseases such as metabolic syndrome [35,72,73,74,75]. Rahati et al. [75] showed a significant difference between *CLOCK* genotypes with a wide range of variables related to human behaviors. Moreover, minor allele *C* carriers in rs1801260 were more of the evening chronotype and tended to sleep less compared to *TT* carriers [35,74]. 

It is known that melatonin is produced when we are sleeping and fasting. The melatonin receptor 1B (*MTNR1B*) SNPs affect melatonin signaling, decrease glucose sensitivity of pancreatic β-cells, and negatively impact circadian insulin secretion [68]. Rs10830963 and rs1387153 in *MTNR1B* were also significantly correlated with gestational diabetes mellitus (GDM), the common metabolic disorder during pregnancy [76,77]. Other evidence for the role of the *BMAL1* gene in the increased risk of glucose intolerance, T2DM, and GDM has been shown [78,79,80]. 

Other several SNPs, including polymorphisms of the *PER2* gene (rs2304672 *C* > *G* and rs4663302 *C* > *T*) and the *Rev-erb-a* gene (rs2314339), have been associated with abdominal obesity, frequent snacking, and skipping breakfast [81,82]. In particular, minor allele carriers *G* of *PER2* rs2304672 displayed several obesogenic behaviors including a decreased of success of the weight loss treatment, increased frequency of snacking, stress while dieting, eating while bored, and skipping breakfast, when compared to carriers *C* [81]. This evidence underlines that individuals carrying specific genetic variants tend to eat more, sleep less, and have greater risk of obesity [83]. To note, these relevant findings are functionally explained since several variants triggering a change in the mRNA structure may lead to a modification in gene expression [84]. 

Nutrigenetics, which is a branch of nutritional genomics, focuses on the role of genetic susceptibility to diseases as well as on the link between genetic variants and response to diet [85,86]. In the era of nutrigenetic research, the relationship between circadian system gene variants and the effectiveness of dietary intervention is noteworthy (Table 1). *CRY1* rs2287161 represents an example of gene–diet interaction for insulin resistance in Mediterranean and North American populations [87]. The *CC* carriers of *CRY1* rs2287161 that ate high amounts of carbohydrates showed higher insulin resistance when compared to *G* carriers whose values of model assessment of insulin resistance (HOMA-IR) were independent of carbohydrate intake, remaining constant [87]. Moreover, other gene–diet interactions associated with MetS at the *CLOCK* locus have been demonstrated. A protective effect of minor allele *CLOCK*-rs4580704 on insulin sensitivity [71] has been shown when MUFA intake was >13.2% of energy. An association between this variant combined with other SNPs in linkage disequilibrium (i.e., rs1801260, rs3736544, rs4864548 and rs3749474) and lower hyperglycemia and decreased risk of T2DM has also been reported [71,88].

Several studies suggested the association between *CLOCK* 3111*T* > *C* (rs1801260) and weight loss effectiveness [74,83,89] showing *C* carriers to be more resistant to weight loss than *TT* homozygotes during an energy-restricted diet [89]. In addition, the *SIRT1* rs1467568 and *CLOCK* 3111T > C combined genotype was associated with the evening chronotype and weight loss resistance in a behavioral therapy treatment for obesity [90]. The authors suggested that the additive effect of *SIRT1* and *CLOCK* variants on resistance to weight loss could be related to the chronotype of the subject, higher plasma levels of ghrelin, and less adherence to Mediterranean diet patterns. Moreover, the deleterious effect of *CLOCK* 3111*T* > *C* on waist circumference was only found with high saturated fatty acid intakes (>11.8%) [71]. Regarding the interaction between *CLOCK* 3111*T*/*C* and emotional eating behaviors to modulate total weight loss in overweight and obese subjects, López-Guimerà et al. [91] showed that minor *C* allele carriers with a high emotional score lost significantly less weight than those *C* carriers with a low emotional score.

These results are encouraging, since by changing our eating habits it is possible to reduce or even eliminate the deleterious effect induced by a specific allele risk. The interplay between gene variants in circadian machinery and diet demonstrated by some intervention studies described above may help to design effective, personalized nutritional strategies based on the identification of specific allele carriers. Further research is required to optimize the individual’s response to the dietary interventions.

**Table 1:** Summary of human studies investigating the interaction between diet and SNPs in circadian clock genes.

Authors (Ref)	Sample Size (*N*)	Study Design	Target Gene (Genetic Variants)	Main Findings
Garaulet et al., 2009 [71]	N = 1100 (540 men, 560 women)	Cross-sectional study	***CLOCK*** rs4580704 *C* > *G* rs3749474 *C* > *T* rs1801260 (3111*T*→*C*) rs1464490 *C* > *T* rs4864548 *G* > *A*	Association with obesity and MetS The minor allele *G* of rs4580704 showed lower risk of hypertension and diabetes. Protective effect the minor allele G of rs4580704 on insulin sensitivity when MUFA intake was >13.2% of energy. Different effects across *CLOCK* 3111*T*→*C* genotypes for saturated fatty acid intake (% of energy) (*p* = 0.017).
Garaulet et al., 2010 [89]	N = 454 Overweight/obese, aged 20 to 65	Dietary program based on the Mediterranean diet (28 weeks)	***CLOCK*** rs1801260 (3111*T*→*C*) rs3749474 *C* > *T* rs4580704 *C* > *G* rs1464490 *C* > *T* rs4864548 *G* > A	Relationship between *CLOCK* SNPs and obesity. *CLOCK* rs1801260 may predict the outcome of body weight reduction strategies based on low-energy diets.
Garaulet et al., 2010a [81]	N = 454 overweight/obese, aged 20 to 65 (380 women, 74 men)	Weight loss intervention based on the Mediterranean diet	***PER2*** rs230467 C > G rs4663302 *C* > *T* rs4663307 *G* > *A*	Association with abdominal obesity (*p* < 0.05) Minor allele carriers G of rs2304672 displayed several obesogenic behaviors. The frequency of the carriers of rs4663307 minor allele who withdrew was greater than in those who successfully completed treatment.
Garaulet et al., 2012 [90]	N = 1106 (715 lean and 391 overweight or obese, aged 20 to 65)	Behavioral treatment for obesity based on a Mediterranean diet (30 weeks)	***SIRT1-CLOCK*** rs1467568 *G* > *A* 3111*T* > *C*	A higher resistance to weight loss and a lower weekly weight loss rate in carriers of minor alleles at *SIRT1* and *CLOCK* loci as compared with homozygotes for both major alleles.
Rahati et al., 2022 [75]	N = 403 overweight and/or obese, aged 20 to 50	Cross-sectional study	***CLOCK*** rs1801260 (3111*T*→*C*)	Significant difference between genotypes for physical activity (*p* = 0.001), waist circumference (*p* < 0.05), BMI (<0.01), weight (*p* = 0.001), GLP-1 (*p* = 0.02), ghrelin (*p* = 0.04), appetite (*p* < 0.001), chronotype (*p* < 0.001), sleep (*p* < 0.001), food timing (*p* < 0.001), energy (*p* < 0.05), carbohydrate (*p* < 0.05), and fat intake (*p* < 0.001).
Lopez-Guimera et al., 2014 [91]	N = 1272 overweight/obese aged 20 to 65	Prospective longitudinal study	***CLOCK*** rs1801260 (3111*T*→*C*)	SNP interacts with emotional eating behaviors for weight loss.
Garaulet et al., 2014a [82]	N = 2414 (1404 Spanish Mediterranean 810 North American populations)	Cross-sectional study	***REV-ERB-ALPHA1*-** rs2314339 *G* > *A*	A lower probability of abdominal obesity in minor allele *A* carriers (OR = 1.5). No significantly association with energy intake but the physical activity was different by genotype. Interaction between the *REV-ERB-ALPHA1* variant and MUFA intake for obesity in the Mediterranean population (*p* = 0.014).
Dashti et al., 2014 [87]	N = 1548 (728 Mediterranean and 820 European origin North American populations)	Cross-sectional study	***CRY*** rs2287161 *G* > *C*	Significant interactions between the *CRY1* variant and dietary carbohydrates for insulin resistance in both populations (*p* < 0.05).
Garaulet et al., 2011 [92]	N = 1495 overweight/obese, aged 20 to 65 years	Cross-sectional study (weight loss program 12–14 weeks)	***CLOCK*** rs1801260 (3111*T*→*C*)	Carriers of the minor *C* allele were more resistant to weight loss, showed shorter sleep duration, higher plasma ghrelin concentrations, delayed breakfast time, evening preference, and less compliance with a Mediterranean diet pattern than *TT* individuals.

MeS: metabolic syndrome, OR: odd ratio, MUFA: monounsaturated fatty acids; BMI: body mass index.

## 7. Epigenetic Alterations in the Clock Genes

The epigenetic mechanisms including DNA methylation, micro-RNAs, and histone modifications, regulate gene expression and control many cellular and physiological processes [85,93,94]. The epigenetic alterations have been considered as potential contributors to the developmental origin of health and disease [95]. Different dietary patterns, lifestyles, and environmental insults are able to modulate the DNA methylation which occurs at the 5′ carbon of cytosines in CpG dinucleotides mainly in gene promoters, and recently the influence of these mechanisms on the circadian rhythm has been reported [96]. In support of this, Azzi et al. [97] suggested that modifications of DNA methylation, the most intensely studied epigenetic mechanism, may have an important role in driving circadian clock plasticity. The authors showed that a transient exposure to a 22 h light–dark cycle induced long-lasting changes in the SCN transcriptome by altering global DNA methylation, which in turn correlated with many behavioral and physiological changes in mice [97]. Apart from the finding described above, this study demonstrated that these changes were relatively stable, but were also reversible after prolonged re-entrainment to the 24-h day. The question of whether the light itself directly regulates the enzyme activity involved in DNA methylation remains to be clarified [98].

Several lifestyle habits involved in the circadian rhythm, such as job seniority, length of shiftwork, and morning and evening types, have been associated with the promoter methylation of the glucocorticoid receptor (GCR), tumor necrosis factor alpha (TNF-α), and interferon gamma (IFN-γ) in blood [99]. 

Hypomethylation in the promotion of the *CLOCK* gene and hypermethylation of *CRY2* in the peripheral blood DNA of subjects on long-term shiftwork have been shown [100]. In addition, DNA methylation patterns at clock genes have been correlated with several outcomes in response to dietary weight loss interventions (Table 2) [101,102,103]. Different DNA methylation levels of several CpG sites of *CLOCK* and *BMAL1* were found between normal-weight and overweight and obese subjects in white blood cells obtained before the 16 weeks weight reduction treatment [101]. In addition, the authors showed significant associations between the methylation in the *CLOCK*, *BMAL1*, and *PER2* with anthropometric parameters such as BMI, adiposity, and MetS score [101]. Moreover, methylation levels of *CLOCK* and *PER2* were associated with several obesogenic behaviors, including snacking frequently and eating when bored, and at baseline were also correlated with the magnitude of weight loss [101]. 

Ramos-Lopez [104] showed associations of DNA methylation profiles at circadian genes (*ROR*, *PRKAG2*, *PER3*, *BHLHE40*, *FBXL3*, *RORA*, *CREB1*) with obesity, metabolic disturbances, and carbohydrate intake, with potential impacts on weight homeostasis in 474 adults.

To date, little is known about the transcriptional regulation of clock genes by the histone modifications. The rhythmic histone H3 acetylation in *mPer1*, *mPer2*, and *Cry1* promoters was reported in the liver and heart, with the peaks occurring during the transcriptionally active phase [105,106]. There are several chromatin modifications that change over the circadian cycle, for example histone H3 serine 10 phosphorylation, the first chromatin mark related to circadian-regulated gene expression identified in mice, increased in the SCN neurons when exposed to light at night [105]. However, some of the details surrounding the histone-modifying enzymes are still underexplored.

Several studies in animal models also suggest that many microRNAs, small non-coding RNA sequences of 22–24 nucleotides, oscillate following a circadian cycle but this is still a poorly explored field of study in humans. A report showed that the microRNA cluster composed of miR-96/miR-182/miR-183 influences the melatonin production, exhibiting diurnal variation in a murine model [107]. In addition, the rhythmic oscillations of miR-96-5p in the regulation of glutathione levels via excitatory amino acid carrier 1 have been demonstrated [108]. Additionally, Zhang et al. 2014 [109] analyzed the circadian expression of genes and non-coding RNAs in different mouse tissues and showed that 39 microRNA levels oscillated in opposite to their target genes. MiR-181, which was previously associated with lipid metabolism, peaked between 8:00 a.m. and 16:00 p.m. in human leukocytes [110]. It has been found that circulating levels of miR-181 decreased in obese subjects, although weight loss normalized its expression. The understanding of the effects of circadian microRNA’s misregulation in human disease is still in its infancy, although several microRNAs influencing the circadian system [111,112] and some of them also modulated by diet, have been considered as potential biomarkers of disease onset and progression [7,113,114]. 

Nevertheless, despite these interesting data, it remains unclear whether the epigenetic changes in the clock genes are causes or effects of obesity and MetS [101]. Moreover, future research should elucidate whether stable changes in eating behaviors may modify the epigenetic mechanisms and consequentially our destiny. In this regard, nutrimiromics, which studies the influence of the diet on the modification of gene expression due to microRNAs [115], and chronobiology should be merged to evaluate the circadian-related microRNAs and their modulation by dietary compounds in order to understand if this relationship may affect the risk of chronic diseases.

## 8. Gut Microbiome

In recent decades, there has been a growing interest in the study of the GM, which is a complex and dynamic population of microorganisms living in the human gastrointestinal tract, and hence it is considered as an auxiliary “metabolic organ” [116,117]. The GM itself, or through interactions with the host, plays a crucial role in the preservation of the mucosal integrity of the intestinal epithelial barrier and in the digestion, metabolism, as well as in the regulation of many hormones’ levels [118]. The main bacterial phyla in healthy individuals are Bacteroidetes and Firmicutes [119]. The GM has a symbiotic relationship with the host [120]. Dietary regimes, food additives, prebiotic and probiotic supplements, food processing, and cooking choices can contribute to shaping the GM [121,122], thus influencing the related immune and metabolic response of the human host. On the other hand, a high-fat diet (HFD) affects the composition of the GM, leading to a drastic reduction in microbial diversity and the Firmicutes/Bacteroidetes ratio, as well as an increases in different bacteria from the Firmicutes phylum [123,124]. Emerging data also demonstrated that the disruption of the circadian system from the host can influence the composition of GM. On the other hand, the gut microbial community can regulate host circadian and metabolic homeostasis, also exhibiting diurnal oscillations [125,126,127]. Although this relationship remains to be clarified, a combination of circadian-clock-dependent and -clock-independent mechanisms has been proposed [10]. Thus, bacterial rhythms typically have a period of 24 h with variations of bacteria during light and dark periods regulated also by melatonin and temperature [128,129]. As proof of day/night rhythms in microbiome composition and metabolic activity, an increase primarily in Bacteroidetes, Verrucomicrobia, but also the opportunistic Enterobacteriaceae during the sleeping/fasting phases has been found, as opposed the Firmicutes peaks during the waking/eating phase which are diet-driven [130]. Several studies demonstrated that circadian disruptions in sleep, diet, and eating patterns impact the diurnal dynamics of GM structure and activity, which may be associated with host metabolic dysfunction and inflammatory pathways leading to an increased risk of metabolic syndrome [10]. Some recent studies have suggested that personalized diets may modify elevated postprandial blood glucose and its metabolic consequences. Zeevi et al. [130], using a machine learning algorithm which integrates multi-dimensional data (such as blood parameters, dietary habits, anthropometrics, physical activity, and GM), accurately predicted personalized postprandial glycemic response to real-life meals. Moreover, these authors demonstrated that personally tailored dietary interventions, based on these predictions, result not only in significantly improved postprandial (postmeal) glycemic responses, but they also report consistent alterations to the GM. Then, Berry et al. [131] developed a different machine learning model that predicted both triglyceride and glycemic responses to food intake. The authors observed inter-individual differences in postprandial metabolic responses to the same meals. The postprandial glycemic predictions were similar to those reported previously [130], although the analysis methods and input features are different. These interesting findings may be informative for the development of population-wide personalized nutrition as a potential strategy for disease prevention.

Nutrients and bioactive compounds of food can modify gut microbial composition and functions, thus several recent strategies based on the manipulation of GM may at least partially consolidate host circadian rhythms. In particular, plant-food-derived fiber and polyphenols can generate bioactive SCFAs, vitamins, and bioamines, which in turn might help resynchronize circadian rhythms, mitigating some of the modern-lifestyle-associated metabolic alterations [132,133,134].

## 9. Conclusions

Balanced nutrition, as well as the synchronization between clear feeding/fasting cycles with clock-regulated metabolic changes, contribute to maintaining circadian rhythms in behavior and physiology [135]. The link between circadian disruption and metabolic disturbance has garnered much attention. A relationship between genetic variants in some of the clock genes with dietary intake, obesity, T2DM, and metabolic risk (MetS)-related variables has been demonstrated [84,92]. On the other hand, gene–diet interactions can modulate the individual predisposition defined by those variants [7]. rs1801260 (3111*T* > *C*) is one of the most relevant SNPs and *C* carriers, which is characterized by sleep reduction, changes in ghrelin values, alterations in eating behaviors and evening preference, and could cause individuals to be more prone to obesity and failure to lose weight [92]. The assessment of risk genotypes of circadian clock genes could provide insight into the link between chronotype and chrono-nutrition, with significant implications for the prevention and treatment of NCDs.

Another point of interest is the effect of several chrono disruptors, such as sleep curtailment, frequent snacking, nocturnal eating, and bright light exposure at night [81], on risk of obesity, on modifications of the clock’s methylation pattern [101], or on changes in transcriptomes [97]. In this view, dietary intake, an important synchronizer, particularly for the peripheral clocks [136], has been associated with DNA methylation levels in the *CLOCK* gene [101], suggesting that some of these CpGs could be used as biomarkers of weight loss response.

On the other hand, promising findings have been recently reported regarding the role of microRNA in circadian regulation. From a nutrimiromics point of view, this research field should be greatly expanded to clarify if the circadian microRNA–diet interactions could be a tool to epigenetically modulate the circadian system altered by chrono disruptors of modern societies.

**Figure 2:**
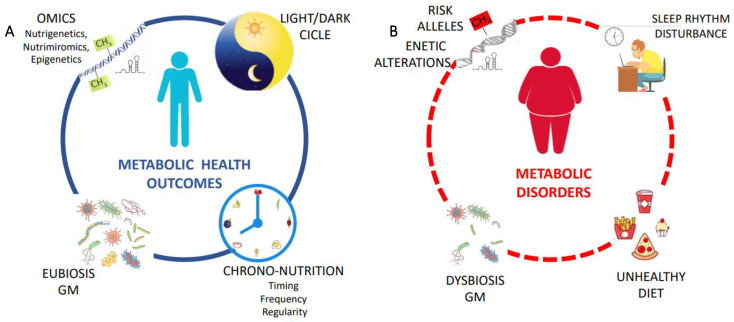
Panel (**A**) From a nutritional genomics perspective, summary of the complex diet–biological rhythm-omics interplay related to health outcomes. Personalized nutrition considering an individual’s genome and epigenome combined with chrono-nutrition could contribute to the fight against non-communicable diseases. Panel (**B**) Diet, chronotype, and several environmental disruptions of modern societies can impact the integration of circadian-triggering metabolic alterations and lead the development of chronic disease mitigation.

Moreover, a dynamic crosstalk exists between GM and the host and recent studies have demonstrated that circadian disruption induced by eating food late at night or at irregular times influences GM, increasing the susceptibility of the host to metabolic dysfunction and inflammation. Manipulating daily rhythms of the microbiome may therefore be a promising chrono-nutrition-based approach to restore the host’s circadian rhythm and metabolic homeostasis [132]. So, considering this, circadian-based strategies have been proposed, such as chronotherapy and food intake only in daylight hours, which might restore the gastrointestinal tract microbiome communities, promoting metabolic health and homeostasis [10,117,137,138]. Therefore, it is possible that prebiotic or probiotic supplements as well as primarily plant-based diets could beneficially alter the microbiota community and circadian rhythms in high-risk populations (i.e., shift workers). Emerging data demonstrated that the clock system influenced by metabolic and epigenetic levels is characterized by remarkable plasticity in response to nutritional challenges. In conclusion, the development of an omics-integral approach based on the knowledge of individual epigenetic and genetic patterns as well as gut microbial composition and activity might provide the basis for personalized nutrition by matching with chrono-nutrition (Figure 2). 

## Figures and Tables

**Table 2:** Summary of human studies investigating the interaction between diet with DNA methylation levels at circadian clock genes.

Authors (Ref)	Sample Size	Study Design	Methylation Profiles in Target Genes	Evidence
Milagro et al., 2012 [101]	N = 20 Normal weight (BMI < 25 kg/m^2^), N = 20 overweight/obese (BMI = 29–35 kg/m^2^) N = 20 morbidly obese women (BMI > 40 kg/m^2^), aged 25 to 53 years	Cross-sectional study (28 weeks of treatment)	*CLOCK* *BMAL1* *PER2*	Association between methylation status of CpG sites located in *CLOCK*, *BMAL1*, and *PER2* with obesity, MetS. The methylation status of different CpG sites in *CLOCK* and *PER2* has been proposed as biomarkers of weight loss success.
Ramos-Lopez et al., 2018 [104]	N = 474 adults belonging to the MENA project	Cross-sectional study	*RORA* *PRKAG2* *PER3* *BHLHE40* *FBXL3* *RORA* *CREB1* *PRKAG2* *PRKAG2*	Correlation between DNA methylation patterns at six circadian rhythm pathway genes with BMI. Correlation between methylation signatures at cg09578018 (*RORA*), cg24061580 (*PRKAG2*), cg01180628 (*BHLHE40*), and cg10059324 (*PER3*) with insulin resistance (*p* < 0.0001) and mean arterial blood pressure (*p* < 0.0001). Relevant correlations between methylation at cg09578018 (*RORA*) and cg01180628 (*BHLHE40*) with total energy and carbohydrate intakes (*p* < 0.05).
Samblas et al., 2016 [102]	N = 61 women (BMI = 28.6 ± 3.4 kg/m^2^; age: 42.2 ± 11.4 years)	Cross-sectional study Weight loss treatment (nutritional program based on a Mediterranean dietary pattern)	*BMAL1* CLOCK NR1D1	The energy-restricted intervention modified the methylation levels of different CpG sites in *BMAL1* and *NR1D1*. The changes in BMAL1 methylation level with the intervention, positively correlated with the eveningness profile (*p* = 0.019). The baseline methylation at *BMAL1* positively correlated with energy (*p* = 0.047) and carbohydrate (*p* = 0.017) intake and negatively correlated with the effect of the weight loss intervention on TC (*p* = 0.032) and low-density lipoprotein cholesterol (*p* = 0.005). Significant and positive correlations were found between changes in methylation levels in the CpG region of *BMAL1* due to the intervention and changes in serum lipids (*p* < 0.05).

MENA: Methyl Epigenome Network Association (MENA) project; BMI: body mass index; TC: total cholesterol.

## Data Availability

Not applicable.

## Abbreviations

SCN: suprachiasmatic nucleus, MetS: metabolic syndrome, SNPs: single-nucleotide, polymorphisms: GM: gut microbiome, SCFAs: short-chain fatty acids, BMI: body mass index, HOMA: homeostatic model assessment, MUFA: monounsaturated fatty acids, HOMA-IR: model assessment of insulin resistance, HFD: high-fat diets, NCDs: non-communicable diseases.
